# Temporary improvement of cognitive and behavioral scales for Dementia elderly by Shiritori word game with a dialogue robot: A pilot study

**DOI:** 10.3389/frobt.2022.941056

**Published:** 2022-12-01

**Authors:** Hiroaki Sugiyama, Kenji Nakamura

**Affiliations:** ^1^ NTT Communication Science Laboratories, Kyoto, Japan; ^2^ Gunma University, Center for Mathematics and Data Science, Gunma, Japan

**Keywords:** Dementia, WOZ test, Shiritori word game, music listening therapy, decreasing BPSD, improving cognitive and behavioral scales

## Abstract

Communication therapies based on conversations with caregivers, such as reminiscence therapy and music therapy, have been proposed to delay the progression of dementia. Although these therapies have been reported to improve the cognitive and behavioral functions of elderly people suffering from dementia, caregivers do not have enough time to spend on administering such communication therapies, especially in Japan where the workforce of caregivers is inadequate. Consequently, the progression of dementia in the elderly and the accompanying increased burden on caregivers has become a social problem. While the automation of communication therapy using robots and virtual agents has been proposed, the accuracy of both speech recognition and dialogue control is still insufficient to improve the cognitive and behavioral functions of the dementia elderly. In this study, we examine the effect of a Japanese word-chain game (Shiritori game) with an interactive robot and that of music listening on the maintenance and improvement of cognitive and behavioral scales [Mini-Mental State Examination (MMSE) and Dementia Behavior Disturbance scale (DBD)] of the dementia elderly. These activities can provide linguistic and phonetic stimuli, and they are simpler to implement than conventional daily conversation. The results of our Wizard-of-Oz-based experiments show that the cognitive and behavioral function scores of the elderly who periodically played the Shiritori game with an interactive robot were significantly improved over the elderly in a control group. On the other hand, no such effect was observed with the music listening stimuli. Our further experiments showed that, in the Shiritori intervention group, there was a ceiling on the increase in MMSE. The lower the MMSE before participating in the experiment, the greater the increase. Furthermore, greater improvement in DBD was observed when the participants actively played the Shiritori game. Since the Shiritori game is relatively easy to automate, our findings show the potential benefits of automating dementia therapies to maintain cognitive and behavioral functions.

## 1 Introduction

Dementia, which frequently occurs in the elderly, is a disease that deteriorates memory and cognitive functions due to organic changes in the brain. These symptoms significantly reduce the elderly’s ability to live independently and maintain quality of life, thus requiring assistance in daily living by caregivers, such as family members and the staff at elderly care facilities (Folstein et al., 1975; Ismail et al., 2010). As cognitive function declines, elderly people with dementia may exhibit problematic behaviors and have difficulty communicating with caregivers (Sugimoto et al., 2018). Among their problematic behaviors, behavioral and psychological symptoms of dementia (BPSD), including hallucinations and violent outbursts, greatly increase the burden on caregivers (Hashidate et al., 2012; Toda et al., 2021). In addition, as communication with caregivers becomes more difficult, the caregiver’s mental burden increases because discrepancies in mutual perceptions are more likely to occur.

Because dementia is accompanied by organic changes in the brain, the disease is never cured completely, even though BPSD and cognitive functions may appear to improve temporarily depending on one’s mood or physical conditions (Cerejeira et al., 2012; Liao et al., 2020). Therefore, when dementia develops, the concern is how to control its progression. Reducing the progression of dementia is expected to maintain the elderly’s quality of life as well as alleviate BPSD, which should reduce the burden on caregivers.

Approaches to reducing the progression of dementia can be broadly classified into pharmacotherapy and non-pharmacotherapy (Hausner and Frölich, 2019). This study focuses on non-pharmacological therapies. Among them, exercise therapy to maintain physical function and reminiscence and music therapies to stimulate memory are commonly used. Exercise therapy can improve physical function through guided intervention by professional staff (Kim and Lee, 2018). Exercise therapy is not intended to directly improve cognitive function but rather to improve physical function, thereby promoting the ability to live independently while maintaining and improving quality of life (Schrum et al., 2019). Reminiscence therapy promotes the activation of memory and increases spontaneous speech through conversations with caregivers or other participants about their own past memories. Spending time to reminisce and talk about the past is expected to stabilize the elderly’s mental condition and thus suppress BPSD (Butler, 1963; Moon and Park, 2020; Morales-De-jesús et al., 2021). Music therapy promotes memory stimulation and spontaneous vocalization, as in reminiscence therapy, by listening to or singing the elderly person’s favorite songs and talking about those songs with the other participants. Music therapy can be categorized as either passive music therapy, in which the elderly simply listen to music, or active music therapy, which involves more active behavior. Passive music therapy is a natural therapy for the elderly whose physical functions have declined.

Although these therapies can be used to reduce the progression of dementia, in practice, the limitation on working hours of qualified staff and caregivers required to implement the therapies has become a major problem. Especially in Japan, where the labor force is decreasing, caregivers do not have enough available time to have adequate conversations with the dementia elderly (Ministry of Health, Labour and Welfare of Japan, 2015). To address this issue, attempts to automate reminiscence therapy using a dialogue system have been reported, but their effectiveness has been limited (Morales-De-jesús et al., 2021). One reason for this difficulty is the inadequate performance of current dialogue systems. Since the elderly with dementia may speak based on thoughts and judgments that differ from those of healthy people, it is difficult for dialogue systems to respond appropriately to such unexpected utterances. In addition, as the muscles associated with speech production decline, articulation becomes degraded, and automatic speech recognition errors are thus more frequent. Due to these problems, improving cognitive function through reminiscence therapy or casual daily conversation with a dialogue system is currently a difficult goal to achieve. Therefore, the application of existing dialogue systems has been limited to very modest targets, such as guidance in exercise therapy or daily medication.

In this study, we focus on a word-chain game as a simple interactive dialogue to activate memory and improve cognitive function in elderly people with dementia (Espen and Gordon, 2015). Since the content of speech in a word-chain game is strongly restricted by rules, it is considered easier to automate than normal conversation. We focus on a traditional Japanese word game, “Shiritori game,” which is commonly played in Japan. It is known to activate dementia elderly participants’ prefrontal cortex (Inoue et al., 2010; Kato et al., 2017). We examine the effect of the Shiritori game with a robot on the maintenance of cognitive and behavioral scales Mini-Mental State Examination (MMSE) (Sugishita et al., 2018) and Dementia Behavior Disturbance scale (DBD) (Mizoguchi et al., 1993) through experiments using a Wizard-of-Oz (WOZ) method. In the Wizard-of-Oz method, a robot controlled by an operator (Wizard) interacts with a participant in such a way that the participant does not perceive that the robot is being controlled. In this way, we can verify the effects of interaction with the robot while compensating for the functions that are currently lacking in autonomous robots. If the game is shown to be useful for improving cognitive function, it can be implemented more easily than conventional interactive robots, so we can expect its early development. Through the WOZ experiment, we collected data on dialogue examples for automation and investigated the necessary behavior of a support robot for dialogue progression.

## 2 Related works

Approaches to reducing the progression of dementia can be broadly classified into pharmacotherapy and non-pharmacotherapy (Hausner and Frölich, 2019). Non-pharmacological therapies, including communication therapy, is the first line and gold standard for the treatment of dementia in the elderly (Kim and Lee, 2018). Pharmacotherapy is mainly used in severe situations due to its harmful side effects, such as delirium (Abraha et al., 2017). Reminiscence therapy is the most effective method of communication therapy using verbal and memory stimulation, which involves sharing each participant’s memories of the past with others. Although reminiscence is a useful method for controlling the progression of dementia, in understaffed nursing homes, it is difficult for qualified caregivers to allocate sufficient time to properly carry it out.

With the aim of increasing conversational opportunities for dementia elderly people, robotic and digital tools are increasingly being used to provide dementia care, and a variety of methods have been proposed. Moon and Park (2020) reported that reminiscence therapy with an app that displays images related to dementia participants’ memories successfully decreased BPSD occurrence in the participants with MMSE scores of 10–19. Morales-De-jesús et al. (2021) developed an interactive virtual avatar that performed automated conversational reminiscence therapy. However, they reported that the performance of its dialogues was insufficient for the participants to enjoy the conversation and to improve their cognitive functions. Continuous daily conversation is still difficult to achieve, even with the recent advances of dialogue systems. Furthermore, natural conversation with dementia elderly people is far more difficult due to their illogical utterances and deteriorated articulation resulting from declined cognitive and physical functions.

Music therapy is another highly effective method frequently used in dementia care. Music therapy is usually performed by combining passive memory stimulation with collaborative work and communication therapy. Gómez Gallego and Gómez García (2017) reported that 42 dementia elderly participants showed improvement in MMSE after 6 weeks of music therapy, in which the participants not only listened to music to stimulate their past memories but also joined music-playing sessions while being encouraged to talk with other participants. Their experiments showed that the MMSE scores of the elderly with mild dementia improved from 18.33 to 22, and those of the elderly with moderate dementia from 12.50 to 17.88. Raglio et al. (2008) also reported that after 16 weeks of music therapy intervention, BPSD in the intervention group was reduced by about 40%. Although music therapy is highly effective, the music listening stimulus is typically combined with accompanying activities such as conversation and singing, which, as with reminiscence, still complicates implementation with autonomous robots. Although music listening stimuli alone can be easily produced by a robot or digital tools, their effectiveness has not been verified.

In this study, we focus on the Shiritori word game, a simple conversational stimulus that can be provided by a robot. The Shiritori game is a commonly used therapy in Japan, along with other non-pharmacological therapies. While Shiritori effectively activates healthy participants’ lower left inferior frontal and language cortex and dementia elderly persons’ prefrontal cortex (Inoue et al., 2010; Kato et al., 2017), it has not yet been examined whether playing Shiritori with a robot can improve the cognitive and behavioral function scales and decrease BPSD of dementia elderly participants.

## 3 Materials

In this study, we examine the effect of a periodic Shiritori game between dementia elderly and an interactive robot, as well as the effect of periodic music listening, on the maintenance and temporary improvement of their cognitive and behavioral scales. To verify the effectiveness of the Shiritori games with a robot precisely and safely, we employed a Wizard-of-Oz (WOZ) method, in which the robot is controlled by a human operator (Wizard) without being noticed by the participants. In the case of validation with autonomous agents, the inadequate accuracy of automatic speech recognition makes validation difficult. In particular, speech recognition accuracy tends to decrease in the case of dementia elderly who have articulation difficulties. In addition, participants suffering from dementia may be more prone to unexpected situations than healthy participants. Even in such unexpected situations, we need to carefully avoid conditions that could be stressful for the participants, and the robot must be able to respond appropriately in these situations. In order to overcome such possible obstacles and to stably verify the effect of the Shiritori game on the maintenance and improvement of cognitive and behavioral function, we adopted the WOZ method in this study.

In this experiment, to handle the possibility of unexpected adverse effects on the participants, the operator controls the robot from a distance where he/she can hear the participant’s voice directly. This also allows the operator to speak directly to the participant if he/she cannot operate the robot in time or if the participant’s attention to the robot’s voice is too weak.

In this section, we describe the details of our Shiritori WOZ system and the songs used for the music listening experiment.

### 3.1 WOZ-operated Shiritori game robot

#### 3.1.1 Shiritori game

The Shiritori game is a traditional word-chain game that is popular in Japan. The following is a description of the rules. First, one of the players of the game says a word that comes to mind. Then the next player says a word that begins with the last letter of that word spoken by the first player. The game proceeds according to the same rules. Usually available words are limited to nouns, and words can be used only once. When one player says a word that ends with a Japanese character “ん,” which is hardly ever the first letter of a Japanese word, he or she loses the game.

#### 3.1.2 System architecture

**FIGURE 1:**
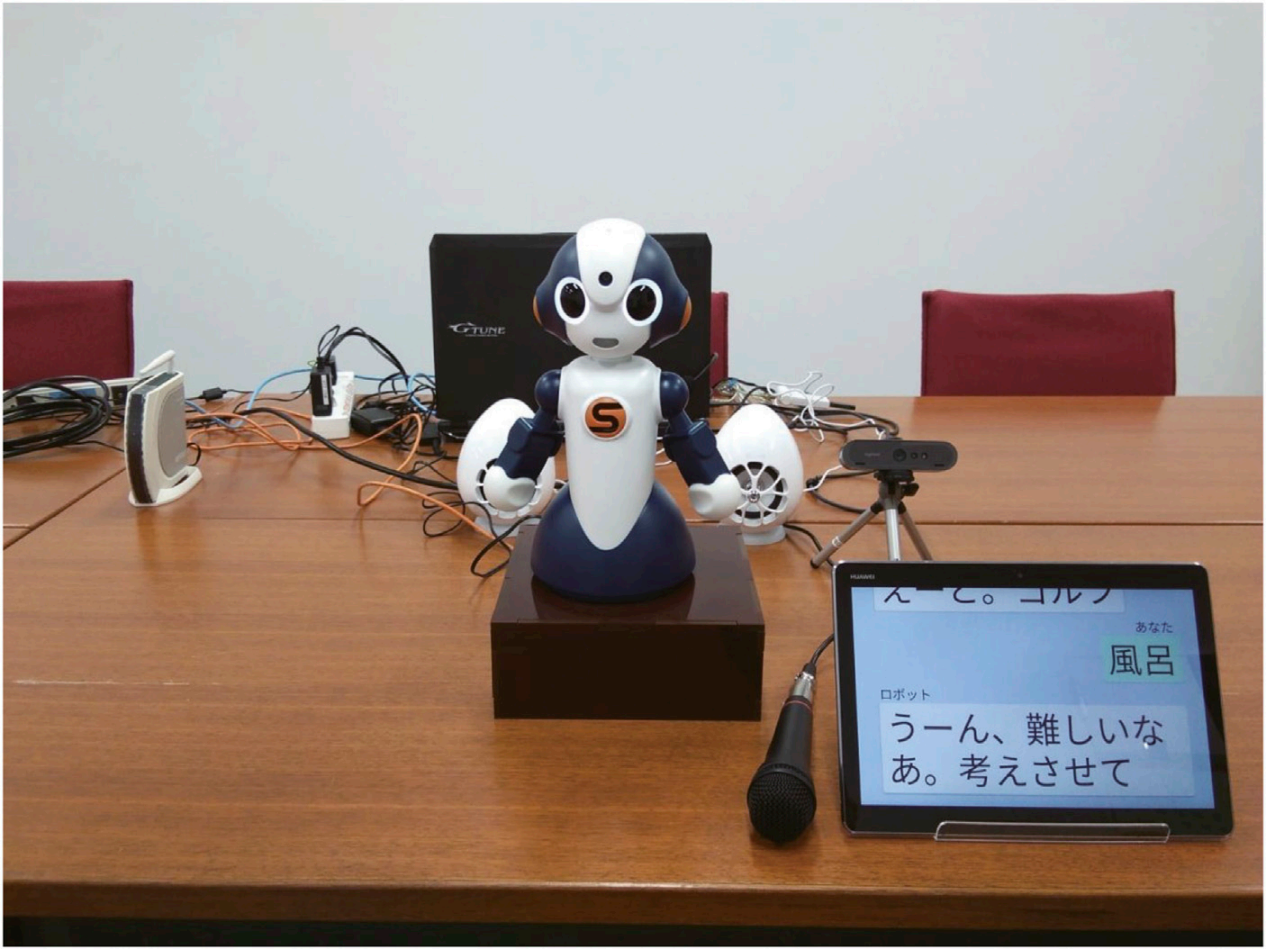
Appearance of WOZ system.

Figure 1 shows the appearance of our Shirotori robot system used in this experiment. A VStone communication robot, Sota^1^, is used as a dialogue agent. The robot has eight degrees of freedom (1 axis for the torso, 2 × 2 for the arms, and 3 axes for the neck), and its eyes can light up when recognizing something or speaking. The robot’s voice is presented using speech synthesis software (FutureVoice) manufactured by NTT-TX.

We use a tablet PC as a sub-display to visually display Shiritori words, speech recognition results, and the robot’s utterances. The user’s speech is input into the system through a microphone and converted into words by the speech recognition system (SpeechRec) manufactured by NTT-TX. Since the WOZ operator directly listens to the participant’s voice, if the recognition results are wrong, he or she modifies them. The operators observe the participants’ behaviors through the video images obtained from a webcam.

#### 3.1.3 Wizard-of-Oz interface

**FIGURE 2:**
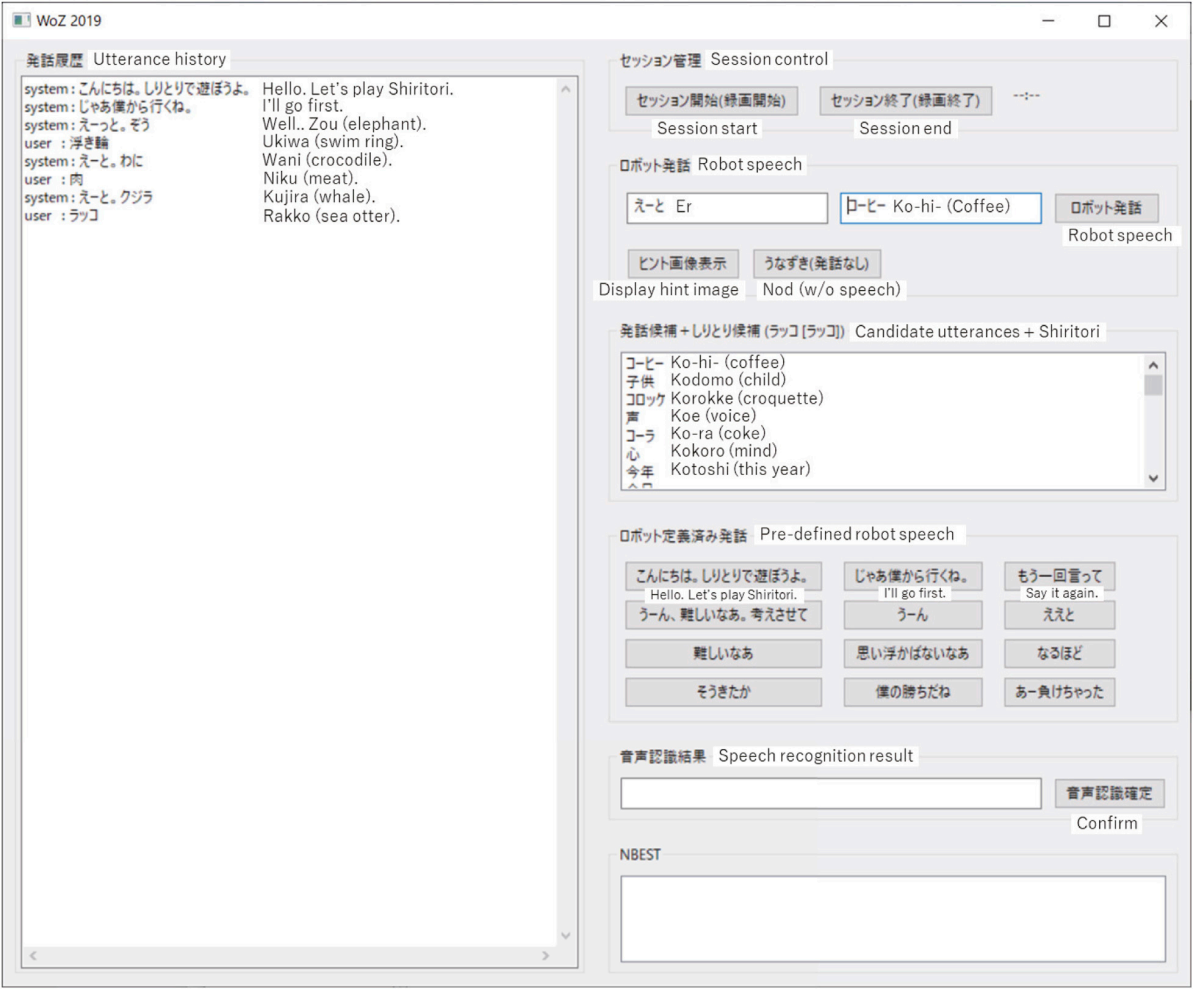
Screenshot of WOZ system interface.

Figure 2 shows the screen of the WOZ system constructed for this experiment. The left half of the screen displays the history of the speech, and the right half is for inputting words. In the lower-left blank area, an image from the webcam was placed so that the user could operate the robot while observing the behavior and facial expressions of the participants in the experiment.

Using the robot’s predefined utterances in the center-right area, the operator can set up the robot to speak a predefined phrase by simply pressing a button. This simplifies the operator’s operation and enables quick responses, as well as collecting training data for making the robot autonomous.

#### 3.1.4 Shiritori game flow with WOZ-operation

The following is a description of the actual Shiritori game flow, together with how the operator exerts control. The WOZ operator first presses the Start Session button on the upper-right side of the WOZ system. This activates the timer on the right side of the button and starts the webcam recording. Next, the operator presses the robot-defined utterances in the middle-right part of the screen to make the robot speak greetings, “Hello. Let’s play Shiritori (こんにちは。しりとりで遊ぼうよ。)” and “I’ll go first (じゃあ僕から行くね。)” in turn. The robot then performs the prescribed actions and synthesizes the speech. The robot speech is also displayed on the tablet PC.

After the greeting, the operator thinks of an appropriate word, types it in the “robot speech” field, and presses the “robot speech” button. When the participant utters a response to the robot utterance, the 1-best recognition result is displayed in the Speech Recognition Result column, and the N-best recognition result is displayed in the NBEST column. The operator also listens to the participant’s voice directly, so he or she can modify the recognition results. By selecting the correct one, or by inputting the correct word in the speech recognition result column and pressing the “confirm speech recognition” button, the speech recognition result is confirmed and displayed on the tablet PC as the result of the person’s utterance.

When the speech recognition result is confirmed, a randomly selected filler utterance is displayed on the left side of the robot utterance column, the first Shiritori candidate word is displayed on the right side, and the remaining candidates of Shiritori words are displayed in the “Candidate utterances + Shiritori” column. Figure 2 shows the screen after the participant in the experiment uttered “sea otter [ラッコ (Rakko)].” As candidates for the next robot utterance, the filler word “er” and the next word “coffee [コーヒー (ko-hi-)]” are displayed. In the “Candidate utterance + Shiritori” column, the top-50 most comprehensible words that do not end in either a muddled sound or “ん” are displayed. If the first word in the Shiritori candidate list (in this case, “coffee”) is difficult to understand, the operator can choose the next word from the list of candidate words. As a measure of understandability, we used a Japanese word-familiarity metric, which is a 5-level measure of how familiar each word is to native Japanese speakers (Amano and Kondo, 1998).

The operator terminates the game when a certain amount of time has elapsed, or when the game is deemed too difficult to continue due to the state of the participant. When the game is terminated due to the lapse of time, the operator intentionally uses words with “ん” to make the robot side lose and terminate the game. In such cases, the robot clearly indicates that it has lost the game by saying, “I can’t think of anything more” and “Oh, I lost the game (負けちゃった).” If the participant’s condition makes it too difficult to continue the game, i.e., if the participant has difficulty in uttering the next word, the robot says, “I win the game (ぼくの勝ちだね)” and ends the game.

During the Shiritori game, the robot says words other than Shiritori words according to the participant’s utterances and situations. If a participant forgets the rules of Shiritori, forgets the situation of playing Shiritori, or does not correctly recognize the previous word, the robot will make a supplementary utterance such as “Say a word starting with X.” If the participant successfully utters a word, the robot will praise the participant’s utterance, such as “Nice choice.” In addition, the robot can also make dialogue-like interjections such as “That’s difficult” or nod when the operator presses the nod button. These utterances are basically pre-defined and uttered when the operator pushes a button on the WOZ interface.

### 3.2 Music listening

In the music listening experiment, participants listen to familiar songs on a periodic basis, and the effects of this activity on their cognitive and behavioral function are examined. The flow of the experiment is as follows. First, we selected songs to be used in the experiment based on interviews with the participants. Each participant was asked to list one to three songs that remain strong in his or her memory. From the candidate songs, we selected the six songs shown in Table 1 so that at least one song chosen by each participant was included. All of them are very famous songs for Japanese of the participants’ generation.

**TABLE 1:** Selected songs for music listening experiment.

No.	Title	Artist	Release year	Sales
1	上を向いて歩こう (Ue wo muite aruko/SUKIYAKI)	Kyu Sakamoto	1961	13.0M
2	川の流れのように (Kawa no nagare no youni)	Hibari Misora	1989	2.1M
3	憧れのハワイ航路 (Akogare no Hawaii kouro)	Haruo Oka	1948	?
4	世界の国からこんにちは (Sekai no kuni kara konnichiwa)	Haruo Minami	1967	3.0M
5	夜霧よ今夜もありがとう (Yogiri yo konnya mo arigato/A Warm Misty Night)	Yujiro Ishihara	1967	2.7M
6	高原列車はいく (Kougen ressya ha iku)	Atsuro Okamoto	1954	?

Using these six songs, a music listening experiment was conducted three times a week. The music data of the six songs were stored on an iPad and made available for selection. At each listening session, participants were asked to select songs from the six songs that were strongly remembered, and the selected songs together had to be longer than 10 min. Participants listened to the selected songs using an iPad. The participants listened to the music alone, with no external intervening voice. In order to investigate the effect of listening to music alone, no video or images were presented. Because no restrictions were placed on the behavior of the participants while listening to the music, the participants exhibited behaviors such as humming the music and swaying their bodies.

## 4 Experiment settings

In this study, we examined the effects of the Shiritori game and of music-listening by examining the changes in cognitive and behavioral function scales between the Shiritori intervention group and the Shiritori control group and those between the music intervention group and the music control group. In addition, for the groups that showed significant differences, we further analyzed in detail the conditions that produced differences in the variation of the cognitive and behavioral function scales.

We conducted the Shiritori game and music listening experiments in different periods. The reason for splitting the experiment into two sessions over time was that the number of participants living in the facility where the experiment was conducted was not large enough to conduct both experiments simultaneously.

### 4.1 Experiment procedure

We conducted the Shiritori game and music listening experiments during different 24-week periods. During each experiment, we tested the cognitive and behavioral scales of the participants every 2 weeks. The first 12 weeks of each experiment were the intervention period, where we examined the effects of each intervention by measuring the difference in scales before and after the intervention as well as the variation trends during the period. After the 12-week intervention period, we followed up the variation of scales with no intervention for 12 more weeks to examine the permanence of the intervention effects.

To examine the effectiveness of the Shiritori game and the music listening in improving the cognitive and behavioral scales, we compared the differences in the scales at week 1 and week 11 between the intervention and control groups. Since the distribution of each difference value was rejected as normal by the Shapiro-Wilk test, we adopted the non-parametric Wilcoxon signed-rank test to verify the differences. We used the Benjamini/Hochberg method to correct for multiple testing, and the corrected *p*-value $\left(\hat{p}\right)$ was used for verification.

#### 4.1.1 Execution of Shiritori game experiments

Participants in the experiment played Shiritori games with the interactive robot for 12 weeks, from 18 November 2019, to 10 February 2020. Each participant played Shiritori games with the robot three times a week, and the length of each game was 10 min, giving consideration to the burden on the participants. The control group only took the cognitive and behavioral scale test and did not participate in the Shiritori experiment, i.e., they did not receive any new instructions about the experiment and continued their usual daily routine.

**FIGURE 3:**
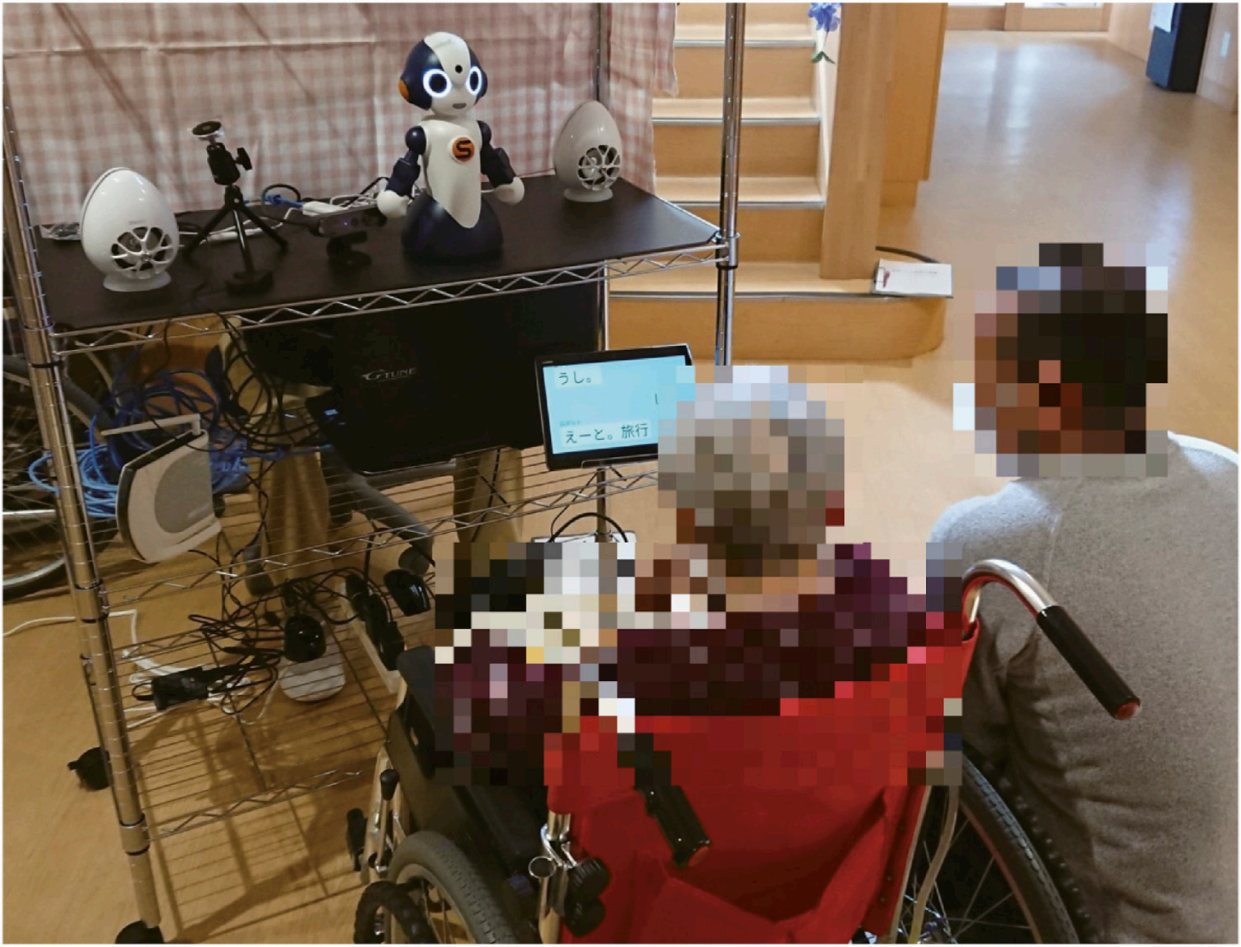
Experimental environment.

In the first week, a supporter (man with masked-out face at the right of Figure 3) guided the participants to play the Shiritori game, but from the second week onward, the game was played without a supporter. If a participant was unable to think of the next word after more than 1 minute, the robot restarted a new game of Shiritori. If the game had to be restarted three consecutive times, the experiment was terminated in consideration of the participant’s mental anxiety. Throughout the experiment, restarts occurred in 45.9% (249/542) of the dialogues, with an average of 1.76 (437/249) restarts per dialogue in which a restart occurred. Termination of the Shiritori game occurred in 2.6% (14/542) of the dialogues.

To measure the effectiveness of the Shiritori intervention, two cognitive and behavioral scale tests (MMSE and DBD scales described in Section 4.3) were performed every 2 weeks. Because the data of the seventh week could not be obtained due to circumstances at the experimental facility, the data for that point were interpolated by using the average values of the preceding and following tests (fifth and ninth weeks).

After the Shiritori game intervention, we continued cognitive and behavioral scale testing for another 12 weeks, the same amount of time as the Shiritori game intervention, and followed up by examining the changes due to this no-intervention condition.

Figure 3 shows a scene of the experiment. We set up the robot in the area where the participants were located 10 min before the start of the dialogue experiment. The robot was set up so that it was at eye level with the participants. The sub-display, which shows the history of the Shiritori game, was set at the height of the participant’s waist. A loudspeaker was placed behind the robot so that the produced sound could be heard smoothly as the robot’s voice. A directional microphone was placed at the same height as the participant’s face. The operators controlled the robot behind curtains near the robot. While the operator could actually be observed by the participants, they perceived the operator as a person who simply watched over the Shiritori game, and they were not aware that the operator was operating the robot. The man with masked-out face at the right of Figure 3 was a supporter, who guided the participants in playing the Shiritori game only in the first week. The operator was a caregiver at the facility, and the supporter was either a caregiver or the co-author.

#### 4.1.2 Execution of music listening experiment

Participants in the music listening experiment periodically took part in music listening sessions for 12 weeks, from 16 November 2020, to 5 February 2021. Each participant performed music listening sessions three times a week, and the length of each session was 10 min considering the burden on the participant. They subjectively selected and listened to songs on an iPad. Since it has been reported that listening to music can have an adverse effect on mental health in rare cases, the facility staff observed the participants from a distance during the experiment to determine whether there were any unnatural symptoms. In the end, no such adverse effects were observed during the investigation, and all participants enjoyed listening to the music. They hummed the tunes, clapped their hands in rhythm, nodded their heads, and recalled the era in which the song was released.

### 4.2 Participants

The participants of the Shiritori game and the music listening experiments consisted of 24 residents of a specific elderly care facility in Gunma Prefecture in Japan. As for the physical care they usually receive, the residents of the facility perform simple exercises in the morning for less than 5 min. Other events such as seasonal events (cherry blossom viewing, *etc.*) and seasonal meals are provided, but dementia care such as reminiscence is not provided other than in this experiment.

#### 4.2.1 Shiritori game validation group

Table 2 shows the basic statistics of the participants of the Shiritori game group. All 24 participants had Alzheimer’s disease, but only one participant in the Shiritori intervention group had vascular dementia. All of the participants consented to the Shiritori game experiment. Since some of the participants might have left the experiment during the several months it was conducted due to the worsening of their medical conditions, to ensure the necessary number of participants for the verification of the temporal changes within the Shiritori intervention group, we had planned to assign a larger number of participants to the Shiritori intervention group than to the control group. Consequently, we randomly assigned 16 of them to the Shiritori intervention group and the remaining 8 to the Shiritori control group. During the intervention period, two of the Shiritori intervention group members left the experiment, so we performed the following experiments with 14 participants in the Shiritori intervention group and 8 in the Shiritori control group. The mean age of the Shiritori intervention group was 88.9 years, and the mean age of the Shiritori control group was 89.1 years.

**TABLE 2:** Statistics of the experiment’s participants. The value of the female column in parentheses is the number of originally assigned participants.

Group name	Female	Male	Age (avg/std)	MMSE of week 1 (avg/std)	DBD of week 1 (avg/std)
Shiritori intervention	12 (14)	2	88.9/4.5	15.9/4.4	23.8/6.25
Shiritori control	7	1	89.1/3.5	15.0/3.5	16.0/10.2
Music intervention	7	1	89.8/5.0	17.2/5.3	23.2/9.2
Music control	5	0	87.4/3.1	18.6/2.5	24.0/1.7

#### 4.2.2 Music listening validation group

The participants in the music listening experiment were 14 individuals who took part in the Shiritori intervention group. We believe that the overlap of participants did not have any impact on the results because we spaced the Shiritori experiment and the music listening experiment over a period of 9 months.

Table 2 shows the basic statistics of the participants of the music listening validation group. All of the participants wished to participate in the music listening experiments. We randomly assigned 9 of them to the music intervention group and the remaining 5 to the music control group. As in the Shiritori game experiment, the music control group was not given any new instructions and was allowed to continue their living routine as usual. No other studies or individual care treatments were provided during the experiment other than the daily 5 min of exercise. The mean age of the music intervention group was 89.8 years, while that of the music control group was 87.4 years.

### 4.3 Evaluation measures

In this study, we used the Mini-Mental State Examination (MMSE) (Sugishita et al., 2018) and the Dementia Behavior Disturbance scale (DBD) (Mizoguchi et al., 1993) as scales to evaluate cognitive and behavioral functions.

The MMSE is a simple and commonly used cognitive function test in clinical settings. It is administered by a person who asks questions of the subject and obtains direct responses on various aspects such as time and place perception, memory, attention, comprehension of verbal and written instructions, and visuospatial cognitive abilities. The maximum score is 30 points, and the lower the score, the worse the cognitive function. A score of 24–27 points indicates suspicion of mild cognitive impairment (MCI), and a score of 23 or less indicates suspicion of dementia.

On the other hand, the DBD scale is designed to quantify BPSD, which is unnatural behavior in the elderly, with higher values indicating more unnatural behavior that causes a greater burden for caregivers.

While we test the cognitive and behavioral scales of the participants every 2 weeks, no training effect affects experimental results. Since the DBD is a third-party evaluation in which subjects’ behavior is observed and examined, no training effect occurs in the subjects. In addition, if there were a learning effect in MMSE, it could be expected that improvement in MMSE would also be observed in the control group.

### 4.4 Detailed analysis factors: Behaviors of the participants and operators in the Shiritori games

In this Shiritori game experiment, we allowed the operators to directly intervene in order to maintain safety during the experiment and to explore the required behavior of an autonomous robot. We examined the frequency of these interventions, the behavior of the dementia participants that caused these interventions, and the relationship between these interventions and changes in their cognitive and behavioral scales. In this study, we examined the correlation between the change in the cognitive and behavioral scales and factors related to the participants’ cognitive status and their behaviors in the Shiritori games, as well as the existence of characteristic groups.

First, we used the cognitive and behavioral scales of week 1 (mmse_w1 and dbd_w1) as an indicator of the participant’s basic cognitive status before the intervention. Since it is naturally assumed that the basic status could affect the subsequent scale changes, we examined the influence of this basic cognitive status on the subsequent improvements.

As behavior in the Shiritori games, we examined the number of utterances by the participants, the frequency of the support utterances, the frequency of the operators’ direct utterances, and the difficulty in situation understanding. The number of utterances by participants (user_utt_count) is an indicator of how many Shiritori utterances they actually made. We examined the relationship between the number of Shiritori game utterances by the participants and the amount of change in the cognitive and behavioral scales.

The frequency of support (support_freq) is the ratio of the number of progression support utterances by the operator over the number of utterances by both the participants and the robots in the Shiritori game experiments. Note that this value does not distinguish between cases in which the robot speaks and cases in which the operator directly speaks. Support utterances are considered to be uttered when the participants have difficulty with the progress of the Shiritori game. Therefore, we also examined the relationship between the difficulty of the Shiritori game progression and the amount of change in the cognitive and behavioral scales.

The frequency of operator utterances in the progression support (operator_support_rate) represents the percentage of operator direct utterances out of the number of the above-mentioned progression support utterances. If this percentage is high, it indicates that the operator’s direct speech is required (i.e., leaving the game or weakening the robot’s attention) to help the participants progress in the Shiritori game, and the robot’s support utterances are insufficient. These conditions mainly compose the state in which the participants’ concentration on the Shiritori game and their intention to continue playing the Shiritori game both decrease. This factor is used to examine the relationship between the occurrence of such situations and changes in the cognitive and behavioral scales. In addition, we also analyzed the effect of the human operator’s direct voice on the scales.

We also defined the state of incomprehension (user_states), which is an indicator of whether the participants did not fully grasp the rules and situation of the Shiritori game. This relates to the MMSE/DBD of week 1, but it directly indicates the difficulty of the participants in continuing the Shiritori game throughout the entire intervention period.

The participants’ and operators’ behaviors in the Shiritori game experiments were annotated by two trained annotators belonging to the same organization as the authors, using the camera video and microphone audio recorded during the Shiritori game. The state of incomprehension was annotated at three levels: 1) Normal, 2) No understanding of the rules, and 3) No response. We used the averaged values of all Shiritori games for each participant (user_states).

## 5 Results

### 5.1 Variations in MMSE and DBD

We examined the differences in MMSE/DBD between the Shiritori game group and the Shiritori control group (Shiritori validation group) and between the music listening group and the music listening control group (music listening validation group).

#### 5.1.1 Shiritori validation group

**FIGURE 4:**
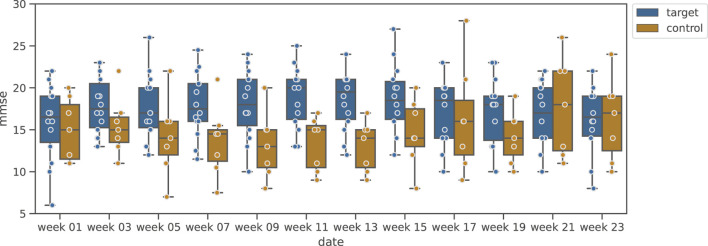
Box plot of MMSE variation in Shiritori validation group.

**FIGURE 5:**
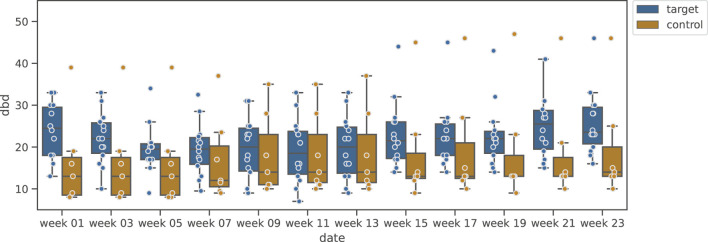
Box plot of DBD variation in Shiritori validation group.

Figure 4 shows the change in MMSE, and Figure 5 shows the change in DBD of the Shiritori validation group. Figure 4 shows that the MMSE score of the Shiritori intervention group gradually increased, with the mean value reaching its maximum at the end of the Shiritori intervention period (week 11). After the intervention, the MMSE gradually decreased and at week 21 reached a lower value than at week 1. The mean MMSE score of the Shiritori control group slightly decreased during the Shiritori intervention period, reaching a minimum at week 9. Similarly, Figure 5 shows that the DBD score (problematic behaviors) of the Shiritori intervention group gradually decreased, reaching its minimum at week 11, and then slightly increased (worsened) and reached a higher (worse) value than week 1 at week 21. On the other hand, the Shiritori control group showed very small fluctuations throughout the 24-week experiment period.

The results of the statistical test are shown in Table 3. For the change in MMSE before and after the intervention (difference between week 1 and week 11), the Shiritori intervention group showed a significantly greater increase compared to the change in the control group $\left(W^{+}=8.0,\;p=1.19e-3,\hat{p}=4.7e-3<.05\right)$ . For DBD, the Shiritori intervention group showed a greater decrease than the change in the control group with a significant trend $\left(W^{+}=23.5,\;p=0.030,\hat{p}=0.060<.1\right)$ .

**TABLE 3:** Significance test of the differences for MMSE and DBD between Week 1 and Week 11 in the Shiritori game and the music-listening experiment.

Stimuli	Measure	Group	Week 01	Week 11	Significance
			(Avg/std)	(Avg/std)	
Shiritori game	MMSE	Intervention group	15.9/4.4	18.9/3.6	*↑*
		Control group	15.0/3.5	13.3/3.0	
	DBD	Intervention group	23.8/6.3	19.1/7.4	(*↓*)
		Control group	16.0/10.2	18.3/8.9	
Music listening	MMSE	Intervention group	17.2/5.3	18.2/4.6	*n*.*s*
		Control group	18.6/2.5	17.8/3.3	
	DBD	Intervention group	23.2/9.2	19.7/11.7	*n*.*s*
		Control group	24.0/1.7	24.2/3.2	

Each value shows the mean and the std of each condition. Higher MMSE and lower DBD indicate better values. Significance arrows indicate *p* < 0.05, and arrows in parentheses indicate *p* < 0.1.

As a qualitative analysis, we conducted a brief interview with the facility’s staff about 1 month after the end of the Shiritori game intervention and obtained the following responses.

• The frequency of violent outbursts decreased. However, after the intervention, the frequency of verbal abuse gradually returned.
• Those with severe emotional transitions became more stable during the intervention period (e.g., crying suddenly decreased). However, previous behaviors returned after the end of the intervention.
• Intuitively, the dementia symptoms stabilized.
• A few days after the Shiritori intervention ended, a few participants asked whether Shiritori games were available or expressed the desire to play Shiritori games, but such behavior disappeared after 2 weeks.
• Some participants sometimes uttered words that sounded like those in the Shiritori game, such as “apple,” but after 2 weeks to a month passed, these behaviors disappeared.

These comments from the interviews show that the effect of the intervention continued for about 2 weeks or 1 month, but it disappeared after this period. These results indicate that playing the Shiritori game periodically with a dialogue robot is effective for temporary improvement of the cognitive and behavioral scales of dementia in elderly people, but the effect disappears within about 4 weeks.

#### 5.1.2 Music validation group

**FIGURE 6:**
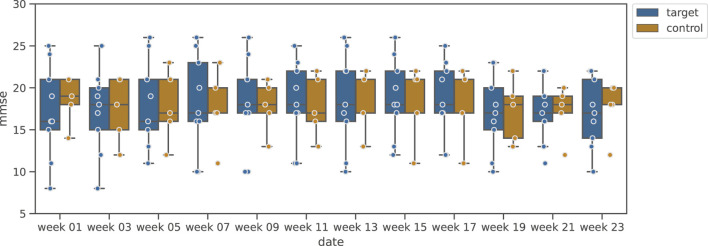
Box plot of MMSE variation in music validation group.

**FIGURE 7:**
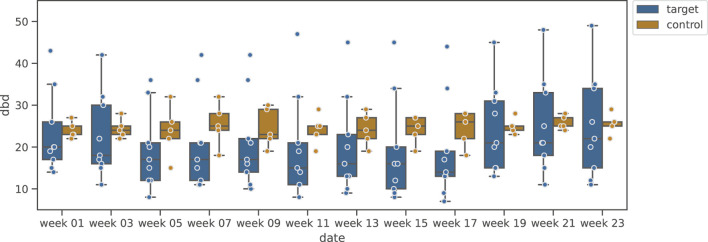
Box plot of DBD variation in music validation group.

Figure 6 shows the variation of MMSE during the music-listening experimental period, and Figure 7 shows the results of DBD variation. Figure 6 clarifies that, unlike the Shiritori intervention group, the music intervention group did not show an increase in MMSE. The variations of the intervention groups are similar to that of the music control group. From Figure 7, the DBD of the music listening group slightly decreased and deviated from the control group, but there was a large variability among participants, where some of them showed high DBD values.

We examined the significance of the change in MMSE and DBD by the music listening intervention based on the significance of the change between week 1 and week 11, as in the Shiritori validation group. The results are shown in Table 3. No significant change was found for either MMSE or DBD (MMSE: $W^{+}=12.0,\;p=0.087,\hat{p}=0.116$ , DBD: $W^{+}=15.0,\;p=0.174,\hat{p}=0.174$ ). These results indicate that music listening, at least, is not sufficiently effective in maintaining or improving the cognitive abilities (MMSE) of dementia elderly people. Although no significance was found for the change in DBD, a trend of decreasing values can be read from the graph. This may be due to the limited power of detection by the small number of participants, which should be verified in a future experiment with a larger number of participants.

### 5.2 Detailed analysis of the differences in the variations

Through the above experiment, we showed that periodic Shiritori games are temporarily effective in maintaining and improving cognitive and behavioral scales and that periodic music listening alone does not produce such effects. Based on these results, this section examines the participants in the Shiritori intervention group in detail and analyzes which factors influenced this difference.

#### 5.2.1 Timeline variations by participants

**FIGURE 8:**
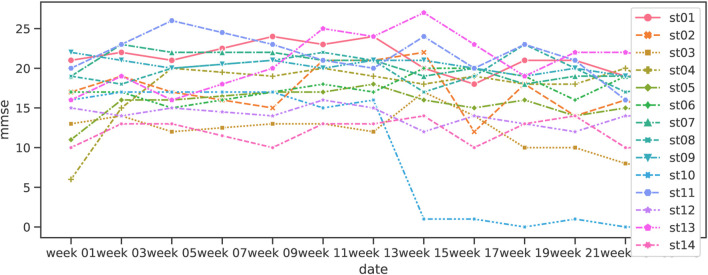
Variation of MMSE in Shiritori intervention.

Figure 8, 9 show the line plots of MMSE and DBD for each participant. The overall increase in MMSE and decrease in DBD during the first 12 weeks is similar to the box plot above, but not all participants show the same trend. In addition, the increase in MMSE was small for those who showed high values in week 1. Considering that the criterion for dementia is MMSE 23 or lower, the ceiling is expected to be approximately 23. On the other hand, the DBD values decreased overall, regardless of the week 1 DBD value, although a few exceptional cases were observed to have an increase in DBD values.

**FIGURE 9:**
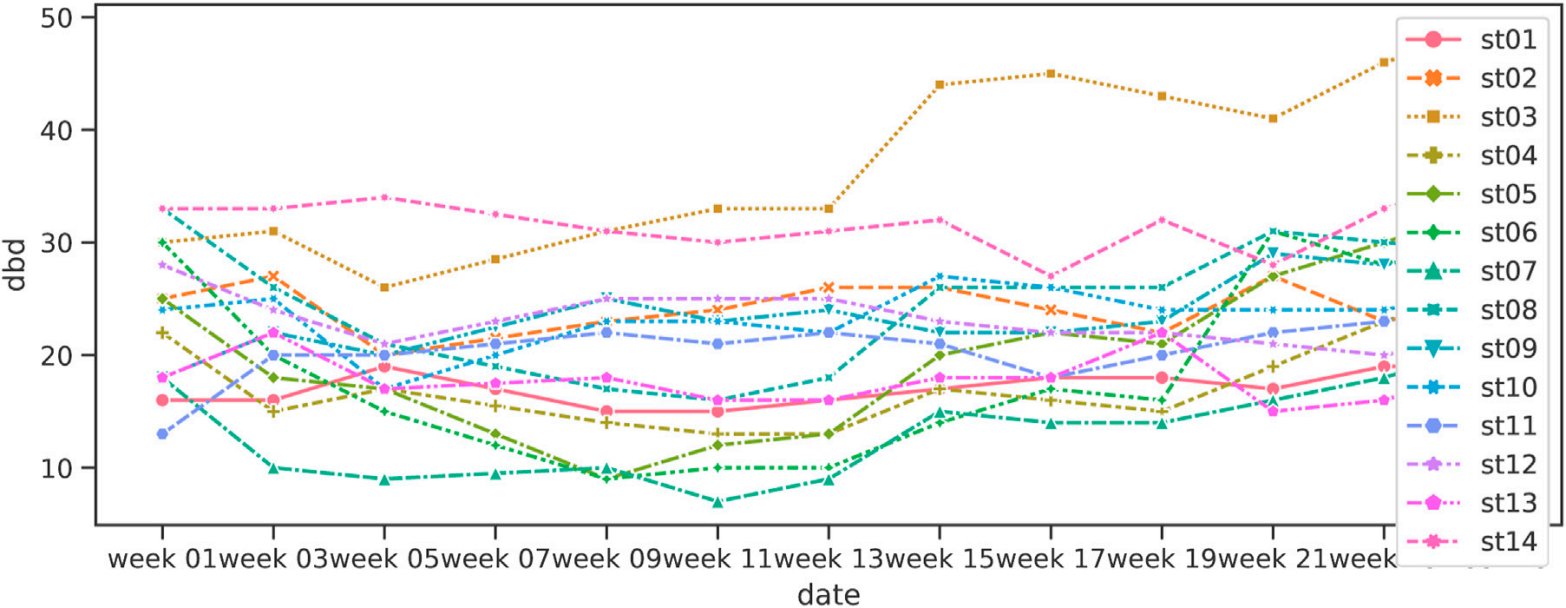
Variation of DBD in Shiritori intervention.

#### 5.2.2 Factors causing MMSE/DBD variations

**FIGURE 10:**
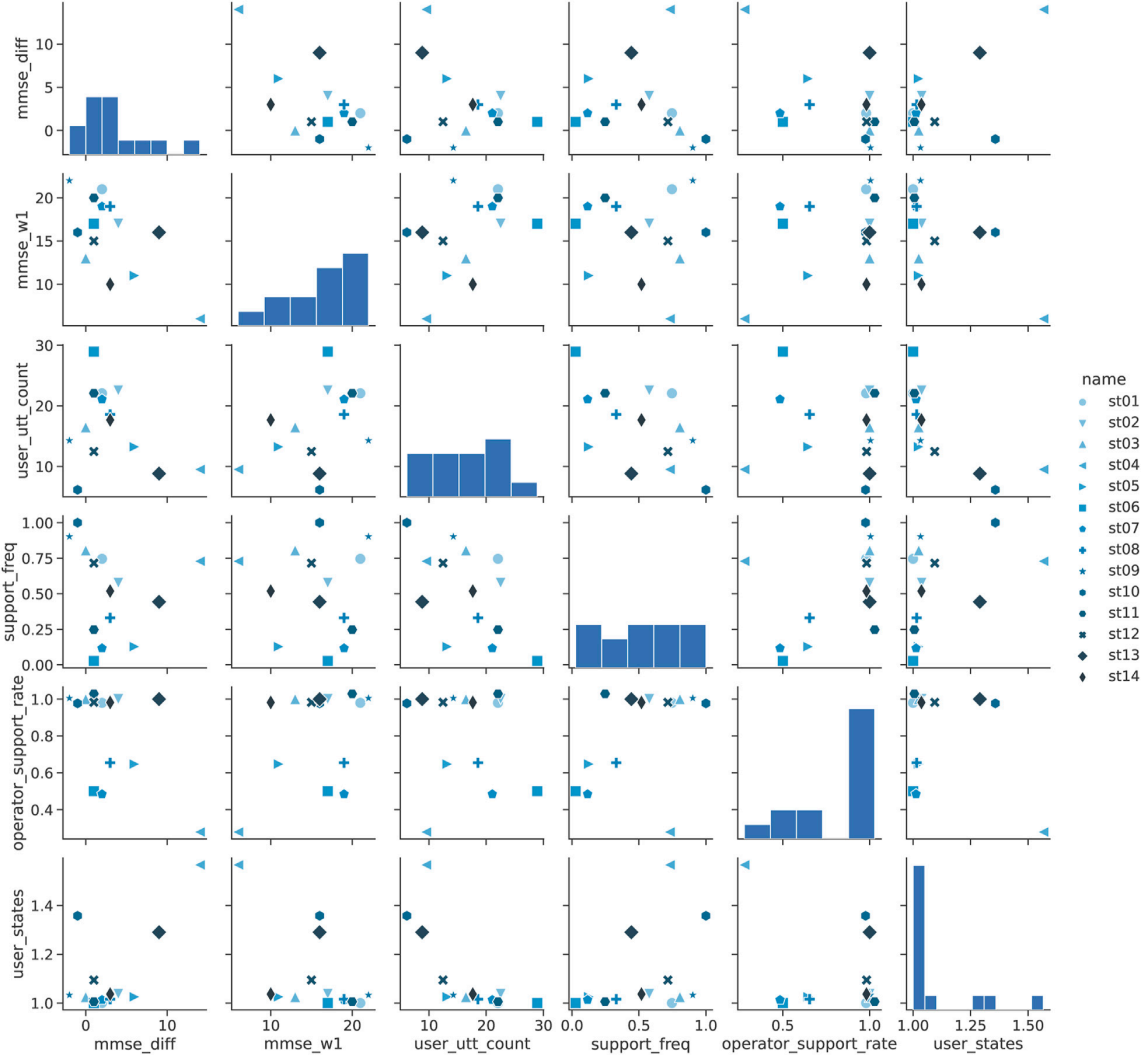
Pairplot analysis of MMSE in Shiritori intervention.

First, Table 4 (a) shows the correlation between these factors and the change in MMSE, and Figure 10 shows a scatter plot of the factors. Table 4 (a) shows that the change in MMSE (mmse_diff) was negatively correlated $\left(r=-0.647,\;p=0.012,\hat{p}=0.032\right)$ with MMSE in week 1 (mmse_w1) and positively correlated $\left(r=0.645,\;p=0.013,\hat{p}=0.032\right)$ with difficulty in understanding the situation (user_state). A negative correlation was also found as a significant trend $\left(r=0.519,\;p=0.057,\hat{p}=0.096\right)$ in the frequency of operator utterances in dialogue progression support (opeartor_support_rate). The scatter plots of these factors show that the MMSE in week 01 (mmse_w1) was negatively correlated with the overall change in MMSE (mmse_diff) and that the increase in MMSE was particularly large in the group with low MMSE in week 01 (st04, st13, st05). This is consistent with the existence of a substantial upper limit of MMSE for dementia participants, and it can be concluded that the change was small except for the participants with a potential for further growth in this increase.

**TABLE 4:** Correlations between the difference in cognitive and behavioral scales between week 1 and week 11 and the factors described in Section 4.4 in the Shiritori intervention group.

	X	Y	r	CI95%	p-unc	p-corr
(a) MMSE						
0	mmse_diff	mmse_w1	−0.647	[−0.88, −0.18]	0.012	0.032
1	mmse_diff	user_utt_count	−0.323	[−0.73, 0.25]	0.261	0.326
2	mmse_diff	support_freq	−0.155	[−0.63, 0.41]	0.598	0.598
3	mmse_diff	operator_support_rate	−0.519	[−0.82, 0.02]	0.057	0.096
4	mmse_diff	user_states	0.645	[0.17, 0.88]	0.013	0.032
(b) DBD						
0	dbd_diff	dbd_w1	−0.483	[−0.81, 0.06]	0.080	0.133
1	dbd_diff	user_utt_count	−0.234	[−0.68, 0.34]	0.420	0.525
2	dbd_diff	support_freq	0.602	[0.11, 0.86]	0.023	0.057
3	dbd_diff	operator_support_rate	0.789	[0.45, 0.93]	0.001	0.004
4	dbd_diff	user_states	0.002	[−0.53, 0.53]	0.994	0.994

Each column title shows that r is the correlation coefficients, CI is the confident intervals of 95%, p-unc is uncorrected *p*-value, and p-corr is corrected *p*-value. Each term indicates the following: mmse/dbd_diff is the difference of MMSE/DBD between week 1 and week 11. mmse/dbd_w1 is the MMSE/DBD scores at week 1. user_utt_count is the averaged number of utterances by the participants in each dialogue. support_freq is the ratio of the number of progression-support utterances by the operator over the number of utterances by both the participants and the robots in the Shiritori game experiments. operator_support_rate is the percentage of operator direct utterances out of the number of progression-support utterances. user_state is an indicator of whether the participants357did not fully grasp the rules and situation of the Shiritori game. The details of each factor is described in Section 4.4.

We analyzed the correlation between the difficulty in understanding the situation (user_state) and the increase in MMSE (mmse_diff) from the scatter plots. Figure 10 shows that only 3 participants had high difficulty, while the other groups showed no difficulty, with the user_state of almost 1.0. Since the increase in MMSE exceeded 7 only when the user_state was high, it is assumed that a significant increase in MMSE was observed in the part of the group that had difficulty grasping even the simple Shiritori game rules, rather than the increase in MMSE being correlated with the user_state as a whole. A more detailed analysis of st10, the only participant in the high-difficulty group whose MMSE did not improve, shows that the number of Shiritori game utterances (user_utt_count) was extremely low. In other words, if the dementia had progressed to the extent that it was difficult to continue Shiritori, it would have been difficult to improve the MMSE by Shiritori intervention.

The frequency of operator speech in the dialogue progression support (operator_support_rate) shows that most participants were around 1.0, indicating that the operator was directly speaking to the participants for some reason. From Figure 10, direct speech utterances become few only in cases where the frequency of supportive utterances (support_freq) is small. It is assumed that, when the operators had to input too many supportive utterances, they became too cumbersome to speak through the robot and thus spoke directly to the participant.

**FIGURE 11:**
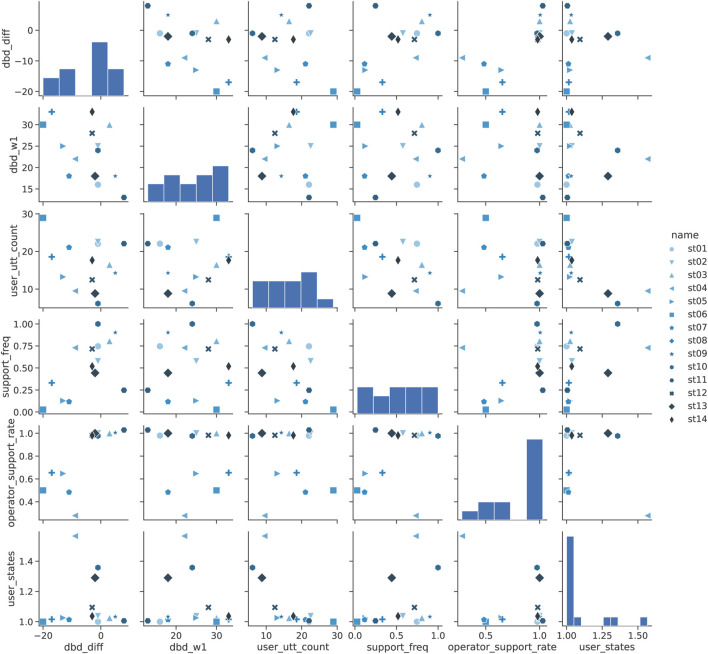
Pairplot analysis of DBD in Shiritori intervention.

DBD is shown in Table 4 (b), and its scatter plot is shown in Figure 11. Unlike MMSE, the difference in DBD (diff_dbd) was strongly correlated $\left(r=0.789,\;p=0.001,\hat{p}=0.004\right)$ with the frequency of operator utterances in dialogue progression support (operator_support_rate) and moderately correlated $\left(r=0.602,\;p=0.023,\hat{p}=0.057\right)$ with the frequency of support (support_freq) as a significant trend. Figure 11 shows that the combination of the frequency of operator utterances (operator_support_rate) and the difference in DBD between week 1 and week 11 (diff_dbd) produced results that were clearly separated into two groups. In other words, there was almost no improvement in the group with the high frequency of operator speech (operator_support_rate), while DBD improved only in the group with the low frequency of operator speech (group in which only robot speech was enough to play the Shiritori game). Considering the fact that there is little association with other factors, we assume that these groups were relatively less in need of support and behaved cooperatively in the progress of the Shiritori game. From these observations, we conclude that DBD improved only among the participants who participated in the Shiritori game in a proactive and cooperative manner.

## 6 Conclusion

In this study, we examined the effect of the Shiritori game with an interactive robot as well as the effect of music listening on the maintenance and improvement of cognitive and behavioral scales (MMSE and DBD) as tools to delay the progression of dementia. The results of experiments on elderly participants with dementia showed that the scales were maintained and improved in the elderly who played the Shiritori games with the interactive robot continuously, compared to the elderly who followed their normal routine. On the other hand, no such effect was observed in the group that listened to music as therapy. In the Shiritori game group, there was a ceiling on the increase in MMSE, and the lower the MMSE before participating in the experiment, the greater the increase. Furthermore, the improvement in DBD was more pronounced in the participants who played the Shiritori game actively and cooperatively.

The future of this project is focused on two main directions. The first is to make the system autonomous. Through the validation of the WOZ method in this study, it was confirmed that playing the Shiritori game with the robot is effective in improving the cognitive and behavioral scales, and thus making the robot autonomous is expected to reduce the burden on nursing home staff. We believe that the autonomous Shiritori robot will be easier to develop than the usual dialogue system targeted by the reminiscence method, and therefore we believe that the robot can be developed at an early stage. For this purpose, the use of the dialogue data obtained in this study is a key issue. Initially, we will verify the degree of speech recognition error using speech data, and then we will improve the speech recognition system using the speech data obtained in this study. In addition, we will improve the accuracy of the automatic selection of appropriate words in the Shiritori game, which was performed by the operator in this experiment, by referring to the operator’s selection log. We also plan to improve the continuity of the Shiritori game played only with a robot voice, even though the operator had to utter direct support utterances in this experiment.

The second future direction is to increase the participants’ active participation in the Shiritori game. Currently, we are planning to conduct experiments in a way that attracts participants’ interest by presenting pictures and other materials as hints, and we will continue such research in parallel with the introduction of the autonomous system. Through these efforts, we aim to introduce the system in a large number of facilities and meaningfully reduce the workload of facility staff.

## Data Availability

The raw data supporting the conclusion of this article will be made available by the authors, without undue reservation.

## Footnotes

1
https://www.vstone.co.jp/products/sota/.
